# Supplementary material for: Prioritising older individuals for COVID-19 booster vaccination leads to optimal public health outcomes in a range of socio-economic settings
Source: PLoS Comput Biol. 2024 Aug 8;20(8):e1012309. doi: 10.1371/journal.pcbi.1012309 (PMC11309497; doi:10.1371/journal.pcbi.1012309)
Supplement: S1 Text — Information about the transmission model and its parameterisation. (PDF) [file pcbi.1012309.s001.pdf]

<u>**S1 Text**</u>

Supplementary details about the epidemiological model

<u>The transmission model</u>

As described in the main text, we used an age-structured model of SARS-CoV-2 transmission and vaccination in our analyses. A schematic illustrating the compartmental structure of the model is shown in Fig 1A in the main text. The model equations are given by:

$$\frac{\mathrm{d}S^{(a)}}{\mathrm{d}t} = -\Lambda^{(a)}S^{(a)} + wS_{W_2}^{(a)}, \qquad \frac{\mathrm{d}S_F^{(a)}}{\mathrm{d}t} = -\nu_F\Lambda^{(a)}S_F^{(a)} - wS_F^{(a)},$$

$$\frac{\mathrm{d}S_B^{(a)}}{\mathrm{d}t} = -\nu_B\Lambda^{(a)}S_B^{(a)} - wS_B^{(a)}, \qquad \frac{\mathrm{d}S_{W_1}^{(a)}}{\mathrm{d}t} = -\nu_{W_1}\Lambda^{(a)}S_{W_1}^{(a)} - wS_{W1}^{(a)} + wS_B^{(a)} + wR^{(a)},$$

$$\frac{\mathrm{d}S_{W_2}^{(a)}}{\mathrm{d}t} = -\nu_{W_2}\Lambda^{(a)}S_{W_2}^{(a)} - wS_{W_2}^{(a)} + wS_F^{(a)} + wS_{W_1}^{(a)}, \qquad \frac{\mathrm{d}E_1^{(a)}}{\mathrm{d}t} = \Lambda^{(a)}S^{(a)} - \alpha E_1^{(a)},$$

$$\frac{\mathrm{d}E_i^{(a)}}{\mathrm{d}t} = \alpha E_{i-1}^{(a)} - \alpha E_i^{(a)} \text{ for } i = 2, \dots, 5,$$

$$\frac{\mathrm{d}E_{1,X}^{(a)}}{\mathrm{d}t} = \nu_X\Lambda^{(a)}S_X^{(a)} - \alpha E_{1,X}^{(a)} \text{ for } X = F, B, W_1, W_2,$$

$$\frac{\mathrm{d}E_{i,X}^{(a)}}{\mathrm{d}t} = \alpha E_{i-1,X}^{(a)} - \alpha E_{i,X}^{(a)} \text{ for } i = 2, \dots, 5 \text{ and } X = F, B, W_1, W_2,$$

$$\frac{\mathrm{d}I^{(a)}}{\mathrm{d}t} = d^{(a)}\alpha E_5^{(a)} - \gamma I^{(a)}, \qquad \frac{\mathrm{d}I_X^{(a)}}{\mathrm{d}t} = \mu_X d^{(a)}\alpha E_{5,X}^{(a)} - \gamma I_X^{(a)} \text{ for } X = F, B, W_1, W_2,$$

$$\frac{\mathrm{d}A^{(a)}}{\mathrm{d}t} = \left(1 - d^{(a)}\right)\alpha E_5^{(a)} - \gamma A^{(a)},$$

$$\frac{\mathrm{d}A_X^{(a)}}{\mathrm{d}t} = (1 - \mu_X d^{(a)})\alpha E_{5,X}^{(a)} - \gamma A_X^{(a)} \text{ for } X = F, B, W_1, W_2,$$

$$\frac{\mathrm{d}R^{(a)}}{\mathrm{d}t} = \gamma\left(I^{(a)} + I_F^{(a)} + I_B^{(a)} + I_{W_1}^{(a)} + I_{W_2}^{(a)} + A^{(a)} + A_F^{(a)} + A_B^{(a)} + A_{W_1}^{(a)} + A_{W_2}^{(a)}\right) - wR^{(a)}.$$

The model parameters are defined in S1 Table. The ν_X parameters reflect the extent to which individuals with different immune statuses are protected from becoming infected compared to a fully susceptible individual, and the μ_X parameters reflect the extent to which individuals with different immune statuses are protected from developing symptoms (conditional on being infected). The force of infection on each susceptible individual in age group a is given by

$$\Lambda^{(a)} = \beta^{(a)}\omega \sum_{b=1}^{16} \frac{M_{ab}}{N^{(a)}} \Big(I^{(b)} + I_F^{(b)} + I_B^{(b)} + I_{W_1}^{(b)} + I_{W_2}^{(b)}$$

$$+ \tau\Big(A^{(b)} + A_F^{(b)} + A_B^{(b)} + A_{W_1}^{(b)} + A_{W_2}^{(b)}\Big)\Big),$$

in which M_{ab} is the average number of contacts that an individual in age group b has per day with individuals in age group a, $N^{(a)}$ is the number of individuals in age group a and τ is the relative infectiousness of asymptomatic individuals compared to symptomatic individuals.

Having numerically solved the model above, we calculated the number of individuals in age group a with different immune statuses (prior to their current infection) that are hospitalised and die on each day t using the following equations:

$$H^{(a)}(t) = P_{IH}^{(a)} \sum_{q=1}^{t} \Delta_q^{IH} J_{t-q}^{(a)}, \quad H_X^{(a)}(t) = \rho_X P_{IH}^{(a)} \sum_{q=1}^{t} \Delta_q^{IH} J_{X,t-q}^{(a)} \text{ for } X = F, B, W_1, W_2,$$

$$D^{(a)}(t) = P_{HD}^{(a)} \sum_{q=1}^{t} \Delta_q^{HD} H_{t-q}^{(a)}, \quad D_X^{(a)}(t) = \phi_X P_{HD}^{(a)} \sum_{q=1}^{t} \Delta_q^{HD} H_{X,t-q}^{(a)} \text{ for } X = F, B, W_1, W_2.$$

Here, Δ_q^{IH} represents the probability that an individual who becomes hospitalised does so q days after entering the I class, and Δ_q^{HD} represents the probability that an individual who dies does so q days after becoming hospitalised. The ρ_X parameters reflect the extent to which individuals with different immune statuses are protected from becoming hospitalised

(conditional on being symptomatic infected) compared to a fully susceptible individual, and the ϕ_X parameters reflect the extent to which individuals with different immune statuses are protected from dying (conditional on being hospitalised).

The value of $J_{F,t-q}^{(a)} = \mu_F d^{(a)} \alpha E_{5,F}^{(a)}(t-q)$ is the rate at which individuals enter the $I_F^{(a)}$ class on day $t-q$. This therefore approximates the number of new symptomatic infections among fully vaccinated individuals arising on day $t-q$. Similar expressions are used for $J_{t-q}^{(a)}$, $J_{B,t-q}^{(a)}$, $J_{W_1,t-q}^{(a)}$ and $J_{W_2,t-q}^{(a)}$.

Model parameterisation

We inferred the values of some of the parameters in our model for the UK initially, due to the availability of surveillance data from that country throughout the COVID-19 pandemic. Previous research with a detailed UK-specific model [1] was used to inform age-dependent weightings for individuals' susceptibility to infection ($\beta^{(a)}$) and the probability that an infected individual develops symptoms ($d^{(a)}$), as well as the relative infectiousness of asymptomatic infectious individuals compared to symptomatic infectious individuals (τ). Using these weightings, we fitted the model to data describing age-stratified numbers of cases, hospitalisations and deaths in the UK between June and September 2022 (a time period corresponding to the third wave of infection after the emergence of the Omicron variant). Specifically, we inferred the values of the infectiousness parameter (ω), the infectious period ($1/\gamma$), and the probability of a symptomatic infected individual developing severe disease ($P_{IH}^{(a)}$) using Markov chain Monte Carlo (MCMC). Considering trace plots for these variables, the MCMC chain was observed to converge after approximately 300 iterations (the first 300 iterations were therefore discarded as burn-in), and the chain was run for a total of 1,000 iterations. To reflect uncertainty, ten combinations of these parameter values were then

sampled from the MCMC results and each used separately in a further fitting process to determine the values of other country-specific parameters.

Characteristics that were assumed to differ between countries include:

- Population age-structure ($N^{(a)}$). The numbers of individuals in each age group were set according to demographic estimates provided by the World Bank [2].
- Contact rates between individuals of different ages (M_{ab}). These were set using country-specific contract matrices reported by Prem *et al.* [3].
- Levels of existing vaccination. Numbers of existing partial, full initial dose and recent booster vaccinations were obtained from Our World in Data [4] and used to inform the initial conditions in our model simulations. When age-based vaccination data were unavailable, previous vaccination was assumed to be applied evenly across adult age groups (individuals aged 18 and over).
- Levels of existing immunity due to previous infection and the probability that hospitalised individuals go on to die ($P_{HD}^{(a)}$). These parameters were determined by fitting to excess mortality data from the Institute for Health Metrics and Evaluation (IHME) [5] up to the end of 2022 using a further MCMC process for each country individually (further details are provided below).

Excess mortality statistics were used in our analyses to reduce the effects of inconsistencies in reporting between countries; excess mortality estimates involve a comparison of death rates in each country during the COVID-19 pandemic with equivalent values in historical data, accounting for past trends and seasonality. While we relied on the IHME's excess mortality estimates, other similar estimates have been generated elsewhere with reasonable consistency; for instance, Karlinsky *et al.* [6] estimated 160,000 deaths in South Africa due to

COVID-19 by 27th June 2021 (60,000 reported) compared to the IHME estimate of 156,373 deaths (with a range accounting for uncertainty of 90,221-258,352).

Each of the ten sets of sampled values for the infectiousness parameter (ω), the infectious period ($1/\gamma$) and the probability of developing severe disease ($P_{IH}^{(a)}$) was then combined with different estimates for excess mortality drawn uniformly at random from within the IHME's confidence intervals for each country [5]. These sets were used in a secondary MCMC fitting process for each country to infer country-specific levels of existing immunity due to previous infection and the overall probability of hospitalised individuals dying ($P_{HD}^{(a)}$). Considering trace plots for all fitted variables, each of these secondary MCMC chains were observed to converge after 300-700 iterations for every country and parameter set. The burn-in period for each chain was chosen to maximise the effective sample size (each burn-in was between 300-700 iterations). A total of 1,400 iterations was deemed sufficient for each chain to represent the target distribution effectively. Ten representative sets of parameters were then sampled at random from each chain (each corresponding to one of the original ten sets of values of ω, $1/\gamma$ and $P_{IH}^{(a)}$ obtained in the initial fitting procedure).

This process thus provided us with a total of 100 parameter sets that were used in all of our subsequent analyses. These comprised of: ten sets of values of the infectiousness parameter (ω), the infectious period ($1/\gamma$) and the probability of a symptomatic infected individual developing severe disease ($P_{IH}^{(a)}$) – all of which are assumed to be identical in all countries – each combined with ten sets per country of country-specific levels of existing immunity due to previous infection and the probability of hospitalised individuals dying ($P_{HD}^{(a)}$). In total, our entire fitting procedure involved running 81 MCMC chains (one chain to infer ω, $1/\gamma$ and $P_{IH}^{(a)}$, and then ten chains per country to infer levels of infection-induced immunity and $P_{HD}^{(a)}$).

The values of the parameters governing the extent to which individuals with infection- or vaccine-induced immunity are protected from infection (e.g. ν_F), developing symptoms (e.g. μ_F), hospitalisation (e.g., ρ_F) and death (e.g., ϕ_F) were calculated from the values shown in Table 1 of the main text. Specifically, the values in Table 1 represent the overall reduction in the risk of each adverse event occurring (e.g. symptom development) for an individual with each immune status compared to a fully susceptible individual. The parameters of the model, however, are conditional on the individual already having entered the relevant compartment of the model.

For example, the parameter μ_F represents the probability that a vaccinated individual develops symptoms, conditional on them having already become infected. We calculated the value of μ_F as follows. First, we noted that if the probability that an unvaccinated individual (with no existing immunity) becomes infected is denoted by x, then based on the values in Table 1, the probability that a vaccinated individual becomes infected is $0.7x$. Conditional on becoming infected, in our transmission model the probability that an unvaccinated individual develops symptoms is d and the probability that a vaccinated individual develops symptoms is $\mu_F d$. Hence, for an unvaccinated individual, the probability of developing symptoms is xd, since symptom development requires both the individual to become infected and develop symptoms. For a vaccinated individual, the probability of developing symptoms is $0.7x\mu_F d$. Since the overall protection against symptom development is 40%, then the relative risk of a fully vaccinated individual developing symptoms (compared to a susceptible individual) is:

$$\frac{0.7x\mu_F d}{xd} = 1 - 0.4,$$

so that $\mu_F = 0.857$. Similar calculations allowed the values of each of the vaccine effectiveness parameters to be obtained from the overall risk reduction values in Table 1. The resulting model parameter values are shown in S1 Table.

References

1. Keeling MJ, Dyson L, Tildesley MJ, Hill EM, Moore S. Comparison of the 2021 COVID-19 roadmap projections against public health data in England. Nat Commun. 2022;13: 4924.

2. The World Bank. DataBank: Population estimates and projections. Available: https://databank.worldbank.org/source/population-estimates-and-projections/

3. Prem K, Zandvoort KV, Klepac P, Eggo RM, Davies NG, Centre for the Mathematical Modelling of Infectious Diseases COVID-19 Working Group, et al. Projecting contact matrices in 177 geographical regions: An update and comparison with empirical data for the COVID-19 era. PLoS Comput Biol. 2021;17: e1009098.

4. Our World in Data. Coronavirus (COVID-19) vaccinations. 2021. Available: https://ourworldindata.org/covid-vaccinations

5. Institute for Health Metrics and Evaluation. Covid-19 mortality, infection, testing, hospital resource use, and social distancing projections. Available: https://www.healthdata.org/node/8660

6. Karlinsky A, Kobak D. Tracking excess mortality across countries during the COVID-19 pandemic with the World Mortality Dataset. eLife. 2021;10: e69336.
