# Supplementary material for: Prioritising older individuals for COVID-19 booster vaccination leads to optimal public health outcomes in a range of socio-economic settings
Source: PLoS Comput Biol. 2024 Aug 8;20(8):e1012309. doi: 10.1371/journal.pcbi.1012309 (PMC11309497; doi:10.1371/journal.pcbi.1012309)
Supplement: S1 Table — The final column indicates whether or not the value of each parameter varies between different parameter sets considered in our analyses. (PDF) [file pcbi.1012309.s002.pdf]

Notation	Meaning	Value	Uncertainty
$\beta^{(a)}$	Infection rate parameter (reflecting the susceptibility of individuals in age group a)	[0.611, 0.354, 0.4, 0.432, 0.608, 0.7, 0.715, 0.69, 0.708, 0.766, 0.896, 1.002, 1.046, 1.307, 1.387, 1.416] per day (different entries correspond to age groups 1,2,…,16)	Fixed
$N^{(a)}$	Number of individuals in age group a	Country-dependent (obtained from [1])	Fixed
M_{ab}	Mean daily number of contacts that an individual in age group b has with an individual in age group a	Country-dependent (obtained from [2])	Fixed
τ	Infectiousness of an asymptomatic infected individual (relative to a symptomatic infected individual)	0.255	Fixed
$1/w$	Period of waning immunity	90 days	Fixed
$5/\alpha$	Mean latent period	5 days	Fixed
$1/\gamma$	Mean infectious period	9 days (mean over all parameter sets)	Varies between parameter sets
ω	Overall infectiousness	0.304 (mean over all parameter sets)	Varies between parameter sets
$\nu_F, \nu_B, \nu_{W_1}, \nu_{W_2}$	Immunity-dependent risk of infection (relative to an unvaccinated individual)	[0.7, 0.6, 0.8, 0.95]	Fixed
$d^{(a)}$	Probability that an unvaccinated infected individual in age group a develops symptoms	[0.068, 0.015, 0.021, 0.026, 0.067, 0.098, 0.104, 0.094, 0.101, 0.125, 0.193, 0.261, 0.293, 0.539, 0.633, 0.678] (different entries correspond to age groups 1,2,…,16)	Fixed
$\mu_F, \mu_B, \mu_{W_1}, \mu_{W_2}$	Immunity-dependent risk of an infected individual developing symptoms (relative to an unvaccinated individual)	[0.857, 0.667, 0.75, 0.842]	Fixed
$P_{IH}^{(a)}$	Probability that an unvaccinated symptomatic infected individual in age group a is hospitalised	[0.0045, 0.0044, 0.0024, 0.0020, 0.0016, 0.0011, 0.0017, 0.0024, 0.0031, 0.0042, 0.0043, 0.0041, 0.0055, 0.0065, 0.0064, 0.0069] (mean over all parameter sets; different	Varies between parameter sets

		entries correspond to age groups 1,2,…,16)	
$\rho_F, \rho_B, \rho_{W_1}, \rho_{W_2}$	Immunity-dependent risk of a symptomatic infected individual being hospitalised (relative to an unvaccinated individual)	[0.286, 0.167, 0.188, 0.421]	Fixed
Δ_q^{IH}	Probability distribution characterising the delay between becoming infectious and being hospitalised	Gamma distribution with mean 0.0001 days and standard deviation 12.1 days. The probability of a delay of q days was assumed to be the probability density of the gamma distribution at value q, normalised for $q = 1,2,\dots,30$ days	Fixed
$P_{HD}^{(a)}$	Probability that an unvaccinated hospitalised individual in age group a dies	Country-dependent, average over fittings and countries: [0.0011, 0.00075, 0.0010, 0.0055, 0.0064, 0.013, 0.012, 0.013, 0.020, 0.022, 0.034, 0.061, 0.10, 0.098, 0.18, 0.30] (mean over all countries and parameter sets; different entries correspond to age groups 1,2,…,16)	Varies between parameter sets
$\phi_F, \phi_B, \phi_{W_1}, \phi_{W_2}$	Immunity-dependent risk of a hospitalised individual dying (relative to an unvaccinated individual)	[1, 0.5, 1, 1]	Fixed
Δ_q^{HD}	Probability distribution characterising the delay between becoming hospitalised and dying	Gamma distribution with mean 10 days and standard deviation 12.1 days. The probability of a delay of q days was assumed to be the probability density of the gamma distribution at value q, normalised for $q = 1,2,\dots,30$ days	Fixed

References

1. The World Bank. DataBank: Population estimates and projections. Available: https://databank.worldbank.org/source/population-estimates-and-projections/

2. Prem K, Zandvoort KV, Klepac P, Eggo RM, Davies NG, Centre for the Mathematical Modelling of Infectious Diseases COVID-19 Working Group, et al. Projecting contact matrices in 177 geographical regions: An update and comparison with empirical data for the COVID-19 era. PLoS Comput Biol. 2021;17: e1009098.
