# Supplementary figures and images for: Prioritising older individuals for COVID-19 booster vaccination leads to optimal public health outcomes in a range of socio-economic settings
Source: PLoS Comput Biol. 2024 Aug 8;20(8):e1012309. doi: 10.1371/journal.pcbi.1012309 (PMC11309497; doi:10.1371/journal.pcbi.1012309)

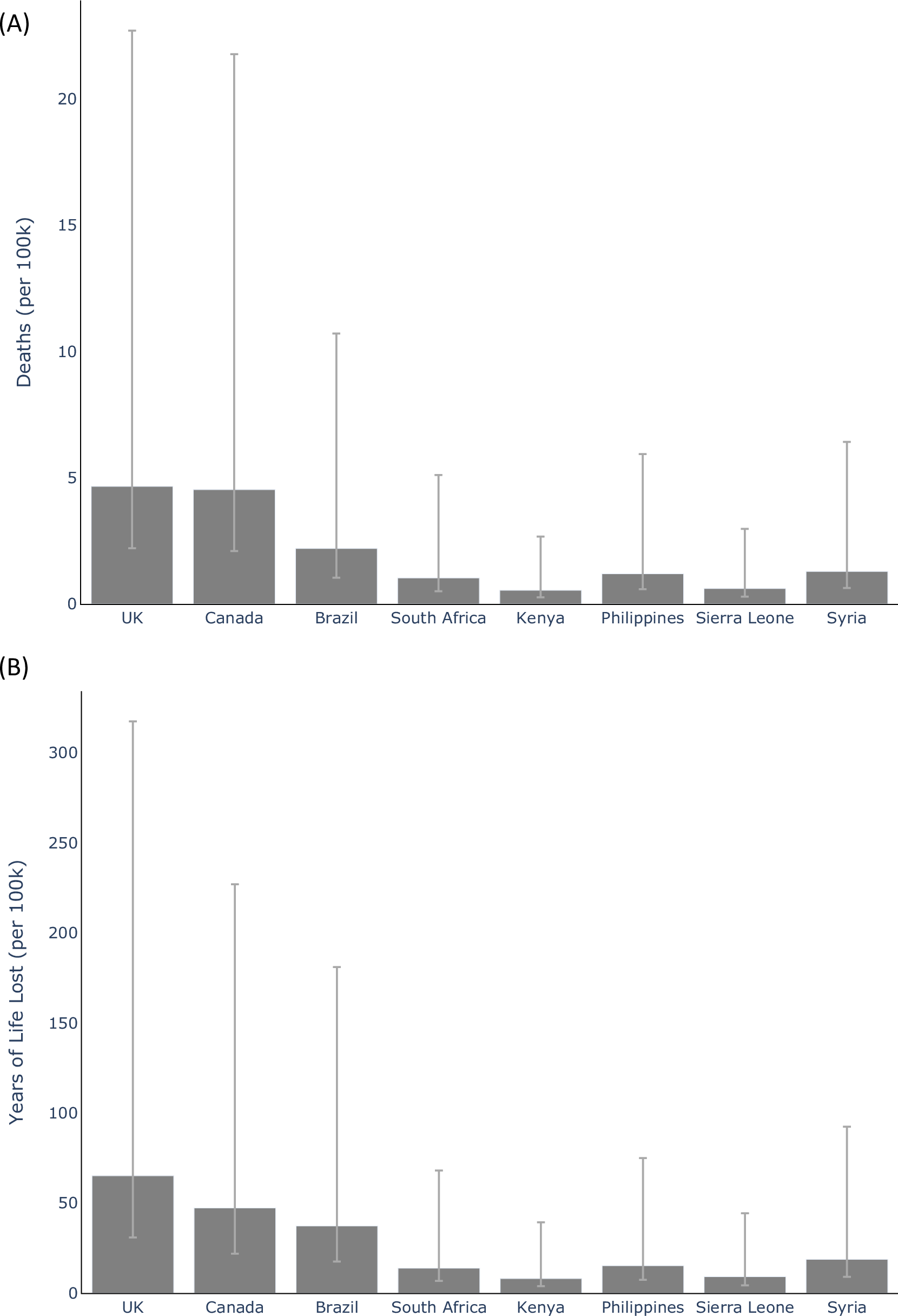

Supplement: S1 Fig — A. The number of deaths (per 100,000 individuals) projected in different countries under booster vaccination Strategy 1. Bars represent the mean values across all parameter sets (cf. yellow bars in Fig 4A in the main text), and lines indicate the 95% prediction intervals (reflecting variation between parameter sets). B. Analogous to panel A, but showing projected YLL rather than deaths. We note that, for any individual parameter set, Strategy 1 leads to the fewest projected deaths and YLL in every country (cf. Fig 4B and Fig 5B in the main text). (TIF) [file pcbi.1012309.s003.tif]

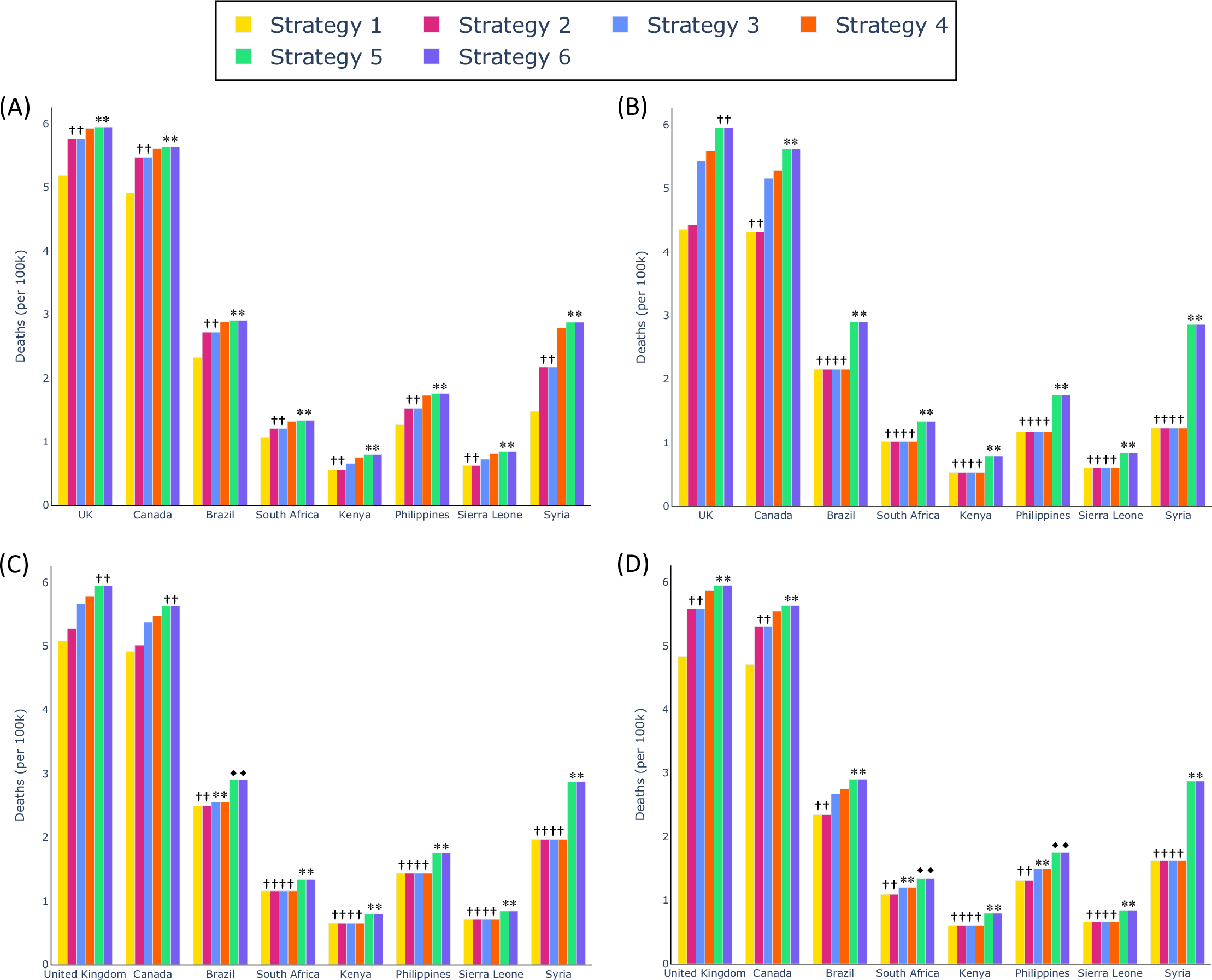

Supplement: S2 Fig — Results are analogous to Fig 4A from the main text, but instead assuming: A. The number of available booster vaccines corresponds to 5% of each country’s population size (rather than 10%). B. The number of available booster vaccines corresponds to 20% of each country’s population size (rather than 10%). C. The maximum uptake of booster vaccination in any age group is assumed to be 50% (rather than 90%). D. The maximum uptake of booster vaccination in any age group is assumed to be 70% (rather than 90%). In each of these scenarios, Strategy 1 led to fewer (or an identical number of) projected deaths than any other booster vaccination strategy. We note that, in some circumstances, different booster vaccination strategies can lead to the same individuals being vaccinated (for example, as shown in Fig 3 of the main text, in our main analyses for Sierra Leone, Strategies 1–4 led to the same individuals receiving booster vaccinations). Here, strategies that led to the same individuals being vaccinated, and therefore identical projected numbers of deaths, are marked within each country and each panel using identical symbols above the relevant bars (for example, in panel A, in the UK strategies 2 and 3 led to the same individuals receiving booster vaccines and strategies 5 and 6 led to the same individuals receiving booster vaccines). (TIF) [file pcbi.1012309.s004.tif]

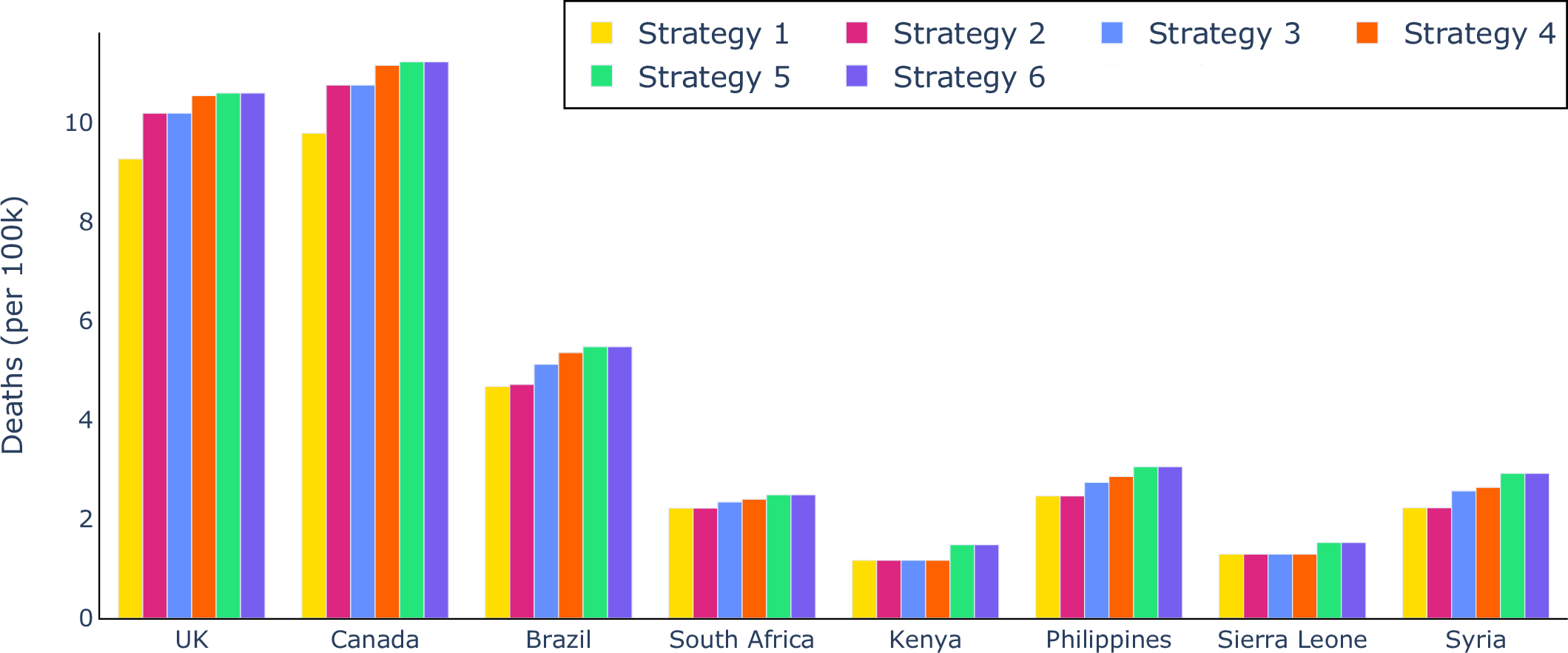

Supplement: S3 Fig — Results are analogous to Fig 4A in the main text, but with a delay of 150 days between booster vaccination and the beginning of the outbreak of the novel variant. The transmission model is first run in the absence of SARS-CoV-2 for 150 days to determine the impact of waning immunity prior to the arrival of the novel variant. The full transmission model (with the novel variant) is then run for a further 150 day period to determine numbers of deaths in different countries. (TIF) [file pcbi.1012309.s005.tif]
