# Supplementary material for: Prioritising older individuals for COVID-19 booster vaccination leads to optimal public health outcomes in a range of socio-economic settings
Source: PLoS Comput Biol. 2024 Aug 8;20(8):e1012309. doi: 10.1371/journal.pcbi.1012309 (PMC11309497; doi:10.1371/journal.pcbi.1012309)
Supplement: Attachment — Submitted filename: cover_letter.pdf [file pcbi.1012309.s006.pdf]

Mathematical Institute
University of Oxford
Oxford OX2 6GG

robin.thompson@maths.ox.ac.uk

Associate Professor of Mathematical Epidemiology

UNIVERSITY OF OXFORD

Mathematical Institute

17 June 2024

Dear Editors,

Prioritising older individuals for COVID-19 booster vaccination leads to optimal public health outcomes in a range of socio-economic settings

Thank you for obtaining these very positive reviews and for provisionally accepting our manuscript. We are grateful to the reviewers for their careful reading of the submission and helpful comments.

We have undertaken the minor changes requested by the reviewers and attach a response in which we itemise and explain our amendments.

Thank you for handling our manuscript, and we look forward to seeing it in print in *PLoS Computational Biology* in due course.

Yours sincerely, on behalf of all authors,

Robin Thompson.

Robin Thompson

Please note: In the material below, whenever we refer to line numbers in the revised manuscript, these refer to the version of the manuscript with “track changes”. We have uploaded two versions of the revised manuscript – one with, and one without, track changes.

Reviewer #1:

The available vaccinations against SARS-CoV-2 protect against severe courses and death from the coronavirus, both on an individual and societal level. However, this protection is not permanent and therefore booster vaccinations are required.

The authors address the important question of which age groups should have access to booster vaccination and in what order to achieve an optimal effect. They consider different scenarios in several countries with the help of a mathematical model.

The methods used are appropriate, rigorous and detailed. The results are novel, relevant, and convincing. The discussion is comprehensive, fair, and balanced.
In approximately 20 years as a reviewer, this is the first time I have recommended accepting a manuscript in its original form. I would like to congratulate the authors on their excellent work.

Response: *We are very grateful to the reviewer for their positive appraisal of our manuscript. We are pleased that they found our methods appropriate and discussion comprehensive, and that the effort that we directed towards this study was worthwhile. We thank the reviewer for their positive comments and encouragement.*

Reviewer #2:

It has been a pleasure to review the manuscript "Prioritising older individiuals for COVID-19-booster vaccination leads to optimal public health outcomes in a range of socio-economic settings".

The authors present a computational study of the outcomes (e.g. years of life lost) of different booster vaccination strategies (in terms of what population groups are prioritised), and apply the approach to different socio-economic settings (from high to low income countries).

The underlying mathematical model is a deterministic, compartmental model of SEIR-type, with a chain of E compartments to model Gamma-distributed exposed times, and age-structure. It is a well-established and well-justified model of COVID-19.

Some of the parameter assumptions are quite strong. For example, assuming that the core infection parameter values are the same in all countries. However, the authors allowed for uncertainty in the parameters, and the results are robust to the uncertainty. However, it is not easy for the reader to discern what parameters are fixed and what

parameters include uncertainty. Similarly, and although the robustness of the results is without question, the details on how some of the parameter models are fitted are not included in the manuscript.

Overall, this is an excellent study, and my comments below are just minor suggestions to improve the readability of the article.

Response: *We are grateful for the reviewer's positive comments and for the suggestions that have allowed us to improve our submission. As noted below, we have added the details requested by the reviewer to the revised manuscript*.

Minor suggestions:

- Include confidence intervals in panel A of figures 4 and 5, even if they are small at that scale. Although the uncertainty is clear from panel B, it will help understand the robustness of the results.

Response: *Thank you for this suggestion. For each parameter set that we considered, Strategy 1 led to fewest deaths and YLL. However, for each individual strategy, there was substantial variation in the number of deaths and YLL between different parameter sets. While showing confidence intervals in panels 4A and 5A would reflect the substantial uncertainty in the precise number of deaths/YLL for each strategy, we are concerned readers might interpret this as suggesting that it is unclear which strategy performs optimally (which, as described above, is not the case; for each parameter set, Strategy 1 is optimal). After substantial reflection about this, we have decided to keep the versions of Figs 4A and 5A from our original submission, but include a new supplementary figure that highlights the substantial variation in projected deaths (Fig S1A in our revised manuscript) and YLL (Fig S1B in our revised manuscript) between parameter sets. We also note that the confidence intervals on Figs 4B and 5B reflect the variation in the performance of each strategy compared to Strategy 1, indicating that the relative benefit of deploying Strategy 1 is very similar between parameter sets.*

We have highlighted the new Fig S1 in the main text (lines 367-368 and 384-387).

- Describe clearly what parameters are taken as fixed, and what parameters include uncertainty.

Response: *We agree with the reviewer that it is important to be clear in our manuscript about which parameter values are fixed, and which parameters account for uncertainty. To address this, we have added a new column to the table of parameters (Table S1) in which we state whether each parameter value is fixed or whether it varies between parameter sets.*

- Describe the details of the MCMC estimates, even if it just in the supplementary material (for example, how many samples were generated, how was convergence assessed).

Response: *As requested, we have added additional details about the MCMC – including the number of iterations run and our approach for assessing convergence – please see lines 63-68 and 99-120 of the revised Supplementary Information.*

- In the supplementary material, Fig S1 suggests that in certain socio-economics settings (e.g. lower income countries), there are no significant differences between several strategies when the assumptions are relaxed (or alternatively, when more uncertainty is allowed in the assumptions). The overall conclusion of the paper in favour of Strategy 1 remains the same, but Fig S1 suggests than in certain settings the policy-makers can take a range of decisions about the strategy without affecting the final outcomes. Please consider including that observation in the main text.

Response: *Thank you for noting that in certain settings, different strategies give rise to identical outcomes. The reason for this is that different booster vaccination prioritisation strategies can sometimes lead to the same individuals being vaccinated. As an example, in a country such as Sierra Leone, with a relatively small population of individuals over the age of 50, then it is possible to vaccinate a large number of individuals aged over 50 irrespective of the order in which individuals aged 50-59, 60-74 and 75+ are prioritised for vaccination. This renders Strategies 1-4 identical in that setting. In contrast, in the UK, where there are large numbers of individuals aged over 50, then a strategy prioritising individuals aged 50-59 will give rise to different vaccinated individuals compared to a strategy prioritising vaccinating individuals aged 60-74, for example (in our simulations, due to our assumption of limited vaccine availability, there simply is not enough vaccine to vaccinate a high proportion of all individuals who are over the age of 50 in the UK). To note clearly which vaccination strategies are identical in Fig S1 (now Fig S2 in the revised manuscript), we have added symbols to the figure to mark strategies with identical outcomes, and explained this in the figure caption.*
